# Olorofim demonstrates *in vitro* activity against *Coccidioides* species, including isolates against which fluconazole has reduced activity

**DOI:** 10.1128/aac.00988-24

**Published:** 2024-11-21

**Authors:** Nathan P. Wiederhold, Hoja P. Patterson, Dora Ferrer, Victor Garcia, George R. Thompson, Thomas F. Patterson

**Affiliations:** 1 Fungus Testing Laboratory, Department of Pathology and Laboratory Medicine, The University of Texas Health Science Center at San Antonio 14742 , San Antonio, Texas, USA; 2 Division of Infectious Diseases, Department of Medicine, The University of Texas Health Science Center at San Antonio 171806 , San Antonio, Texas, USA; 3 Department of Medical Microbiology and Immunology, University of California Davis 272916 , Davis, California, USA; University Children's Hospital Münster, Münster, Germany

**Keywords:** olorofim, *Coccidioides *species, *in vitro *susceptibility, fluconazole

## Abstract

We evaluated the *in vitro* activity of olorofim against *Coccidioides* species. Olorofim demonstrated potent *in vitro* activity against all isolates tested with a minimum inhibitory concentration (MIC) range ≤0.008–0.06 µg/mL and geometric mean MIC of 0.010 µg/mL. This activity was also maintained against isolates with elevated fluconazole MICs (≥16 µg/mL), including strains with MICs ≥32 µg/mL (olorofim MIC range ≤0.008–0.06 µg/mL and geometric mean MICs of ≤0.009 and ≤0.013 µg/mL, respectively).

## INTRODUCTION

Coccidioidomycosis is a fungal infection that is caused by dimorphic, saprobic fungi, *Coccidioides* species, that are endemic to areas of Arizona, California, New Mexico, Utah, Nevada, and Texas within the United States, as well as parts of Mexico, Guatemala, Honduras, Venezuela, Brazil, Argentina, and Paraguay (1–3). Although *Coccidioides* species are found in warm, arid climates, disease can occur outside of these regions in individuals who have visited or temporarily relocated to these regions. In addition, the endemic area of the United States appears to be spreading both northward and eastward, with some predicting that the endemic area may double by 2100 due to climate change (4–6).

Clinically available treatment options for patients with coccidioidomycosis remain limited. The azoles itraconazole and fluconazole are primarily used, and fluconazole remains the preferred triazole due to its excellent absorption following oral administration, low adverse effect profile, penetration into the central nervous system (CNS), and relative affordability as a generic medication (7, 8). However, there is concern for reduced *in vitro* susceptibility of *Coccidioides* isolates to fluconazole (9, 10). In a retrospective review of the *in vitro* susceptibilities of various antifungals against clinical isolates, members of our group reported that over a third of the isolates had fluconazole minimum inhibitory concentrations (MICs) of ≥16 µg/mL (9)—an MIC value in other fungi typically associated with dose-dependent susceptibility or resistance to this triazole, although data correlating *in vitro* results against *Coccidioides* with clinical outcomes are lacking. Olorofim (formerly F901318) is an investigational antifungal within the orotomide class that interferes with the biosynthesis of pyrimidine within fungi through the inhibition of the dihydroorotate dehydrogenase enzyme (11). This agent is active against many genera of pathogenic molds and dimorphic fungi, including *Blastomyces*, *Histoplasma*, and *Coccidioides* species (11–16). We have previously reported that olorofim has good *in vitro* against a limited number of *Coccidioides* clinical isolates, and this translated into *in vivo* efficacy in a murine model of CNS coccidioidomycosis (15). Here, we report the *in vitro* activity against a larger collection of clinical isolates, including those for which fluconazole has reduced *in vitro* activity against as evidenced by elevated MICs.

Clinical isolates of *Coccidioides* species (*n* = 201), including both *C. immitis* and *C. posadasii*, sent to the Fungus Testing Laboratory at the University of Texas Health Science Center at San Antonio from institutions across the U.S. were used. Antifungal susceptibility testing was performed by broth microdilution methods as described in the Clinical and Laboratory Standards Institute (CLSI) M38Ed3 document (17). The starting inoculum was 1 × 10^4^ arthroconidia/mL, and RPMI-1640 [0.165M 3-(N-morpholino) propanesulfonic acid (MOPS), pH 7.0, without bicarbonate] served as the growth medium. Stock solutions at 100× concentrations of olorofim (F2G, Ltd), amphotericin B, fluconazole, posaconazole, voriconazole, itraconazole, and isavuconazole (Sigma) were prepared in dimethyl sulfoxide (DMSO) with further dilutions made in RPMI such that the final DMSO concentration within the *in vitro* assay was 1% (vol/vol). The concentration ranges tested were 0.008–4 µg/mL for olorofim, 0.125–64 µg/mL for fluconazole, and 0.03–16 µg/mL for the other azoles. MICs of olorofim, amphotericin B, posaconazole, voriconazole, itraconazole, and isavuconazole were read at 100% inhibition of growth compared to drug-free control after 48 hours of incubation at 35°C, and fluconazole MICs were measured at 50% growth inhibition. MIC ranges, MIC values at which 50% and 90% of the isolates were inhibited (MIC_50_ and MIC_90_, respectively), geometric mean (GM) MIC, and modal MIC values were determined. Differences in GM MICs, calculated following log_2_ transformation of individual MIC values, were assessed for significance by ANOVA with Dunnett’s post-test for multiple comparisons. MIC values greater than the highest concentration tested were assigned a value one dilution higher for statistical purposes. A *P*-value of <0.05 was considered statistically significant. Fluconazole was considered to have reduced *in vitro* activity against strains with MICs of ≥16 µg/mL.

**Fig 1:**
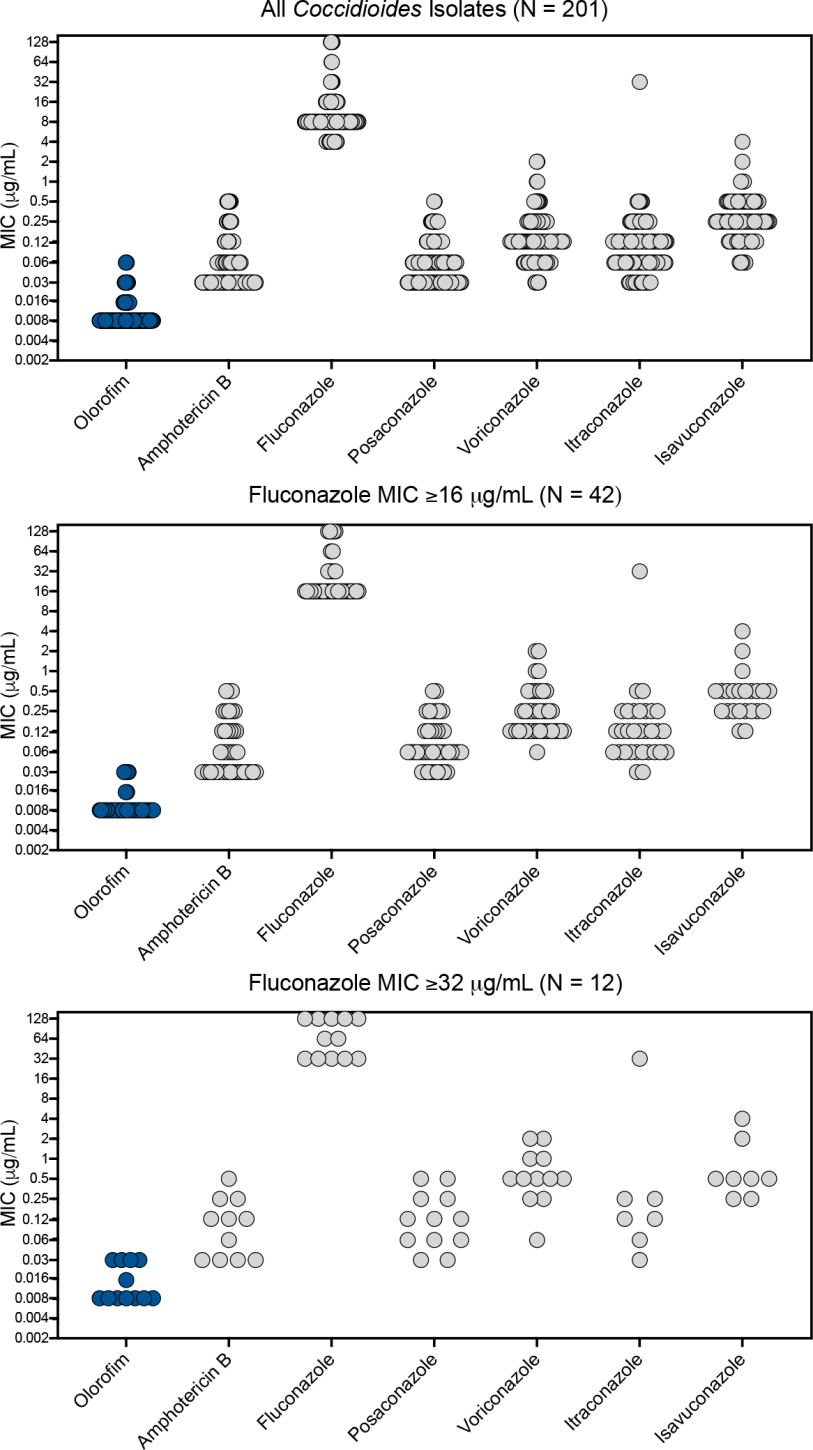
MIC distributions of olorofim, amphotericin B, fluconazole, posaconazole, voriconazole, itraconazole, and isavuconazole against *Coccidioides* species isolates. Results are shown for all isolates tested and those with higher fluconazole MIC values (≥16 µg/mL and ≥32 µg/mL, respectively).

Of the agents tested, olorofim demonstrated the most potent *in vitro* activity with a GM MIC value of ≤0.010 µg/mL, followed by amphotericin B (≤0.051 µg/mL), posaconazole (≤0.054 µg/mL), itraconazole (≤0.095 µg/mL), voriconazole (≤0.128 µg/mL), isavuconazole (0.261 µg/mL), and fluconazole (9.18 µg/mL). The olorofim GM MIC values were significantly lower than those of all other antifungals (*P* < 0.0001 for all comparisons). The enhanced *in vitro* potency of olorofim is also reflected by the MIC_50_, MIC_90_, and modal MIC values, which were numerically lower than those of the other antifungals (Table 1). The MIC distributions are graphically shown in Fig. 1.

**TABLE 1:** MIC of olorofim (OLO), amphotericin B (AMB), fluconazole (FLC), posaconazole (PSC), voriconazole (VRC), itraconazole (ITC), and isavuconazole (ISC) against *Coccidioides* species isolates^*a*^

Antifungal	OLO	AMB	FLC	PSC	VRC	ITC	ISC
All isolates							
No. of isolates	201	196	201	200	200	138	91
Range	≤0.008–0.06	≤0.03–0.5	4–>64	≤0.03–0.5	≤0.03–2	≤0.03–>16	0.06–4
MIC_50_	≤0.008	≤0.03	8	0.06	0.125	0.125	0.25
MIC_90_	0.015	0.125	16	0.125	0.25	0.25	0.5
GM MIC	≤0.010	≤0.051	9.30	≤0.055	≤0.128	≤0.097	0.261
Mode	≤0.008	≤0.03	8	≤0.03	0.125	0.125	0.25
Fluconazole MICs ≤8 µg/mL							
No. of isolates	159	159	159	159	159	139	91
Range	≤0.008	≤0.03–0.06	4–8	≤0.03–0.06	≤0.03–0.25	≤0.03–>16	0.06–4
MIC_50_	≤0.008	≤0.03	8	≤0.03	0.125	0.125	0.25
MIC_90_	≤0.008	0.06	8	0.06	0.125	0.25	0.5
GM MIC	≤0.008	≤0.037	7.14	≤0.041	≤0.098	≤0.097	0.261
Mode	≤0.008	≤0.03	8	≤0.03	0.125	0.125	0.125
Fluconazole MICs ≥16 µg/mL							
No. of isolates	42	41	42	42	42	32	24
Range	≤0.008–0.06	≤0.03–0.5	16–>64	≤0.03–0.5	0.06–2	≤0.03–>16	0.125–4
MIC_50_	≤0.008	≤0.03	16	0.06	0.25	0.125	0.5
MIC_90_	0.03	0.25	>64	0.25	1	0.5	2
GM MIC	≤0.009	≤0.066	23.8	≤0.079	0.234	0.138	0.417
Mode	≤0.008	≤0.03	16	≤0.06	0.125	0.06	0.5
Fluconazole MICs ≥32 µg/mL							
No. of isolates	12	11	12	12	12	7	8
Range	≤0.008–0.06	≤0.03–0.5	32–>64	≤0.03–0.5	0.06–2	≤0.03–>16	0.25–4
MIC_50_	≤0.008	0.125	64	0.125	0.5	---	---
MIC_90_	0.03	0.5	128	0.5	2	---	---
GM MIC	≤0.013	≤0.090	>64	≤0.116	0.528	0.247	0.648
Mode	≤0.008	≤0.03	32	≤0.06	0.5	0.125	0.5

^*a*^ MIC values (mg/mL) were measured according to CLSI M38Ed3 guidelines as the lowest concentration of each agent, except fluconazole, which resulted in 100% inhibition of growth compared to growth control. Fluconazole MICs were read at 50% growth inhibition. MIC_50_ and MIC_90_ – MIC concentrations at which 50% and 90% of the isolates were inhibited.

Olorofim also maintained *in vitro* potency against 42 isolates with elevated fluconazole MICs (olorofim GM MIC ≤ 0.009 µg/mL), including 12 isolates with fluconazole MICs ≥ 32 µg/mL (olorofim GM MIC ≤ 0.013 µg/mL). The GM MIC of olorofim was also significantly lower than those of posaconazole, voriconazole, itraconazole, and isavuconazole (*P* < 0.0001 for all comparisons; Table 1). Although the MICs of the extended-spectrum azoles remained relatively low against isolates with fluconazole MICs ≥ 32 µg/mL, the GM MIC values of each of these azoles against these strains were higher compared to those of isolates with lower fluconazole MIC values (GM MIC range 0.116–0.648 μg/mL vs 0.041–0.261 μg/mL; fold-change 2.48–2.83) and were higher than those of olorofim (*P* < 0.0001). Not unexpectedly, amphotericin B also demonstrated potent *in vitro* activity against those isolates with elevated fluconazole MICs.

These results are consistent with those we previously reported against a smaller number of isolates. In the earlier study, the olorofim GM MIC was 0.011 µg/mL, and all strains were inhibited at olorofim concentrations of ≤0.06 µg/mL. The current study is not without limitations. We did not confirm the species level (*C. immitis* vs *C. posadasii*) for all isolates tested. However, previous work reported by members of our group has not demonstrated difference in *the in vitro* activity of fluconazole, voriconazole, or olorofim between these two species when tested against a limited number of strains (15, 18, 19). Also, the concentration ranges used for olorofim, amphotericin B, and posaconazole were not low enough to accurately measure the *in vitro* activity of these agents. Thus, these three agents may have more potent activity than demonstrated here. The ranges used for amphotericin B, posaconazole, and the other azoles were consistent with those recommended in the CLSI M38 standard (17). The range recommended for olorofim has not yet been established. Further work is also needed to understand the mechanisms underlying the elevated fluconazole MICs observed against some strains.

Olorofim is currently in late-stage clinical development and has been used to treat a limited number of patients with coccidioidomycosis who have failed to respond to clinically available antifungals. In an open-label phase 2 study, 75.6% of patient with extrapulmonary coccidioidomycosis demonstrated clinical benefit by day 42 of olorofim treatment and 73.2% by day 84 (20). Further studies are warranted to fully understand the utility of olorofim against *Coccidioides* infections, including infections caused by isolates with reduced azole susceptibility.
